# Chitosan/Cyclodextrin/TPP Nanoparticles Loaded with Quercetin as Novel Bacterial Quorum Sensing Inhibitors

**DOI:** 10.3390/molecules22111975

**Published:** 2017-11-15

**Authors:** Hao Thanh Nguyen, Francisco M. Goycoolea

**Affiliations:** 1 Institute for Biology and Biotechnology of Plants, University of Münster, Schlossgarten 3, 48149 Münster, Germany; t_nguy32@uni-muenster.de or nthao.cnsh@vnua.edu.vn; 2 Department of Biology, Faculty of Biotechnology, Vietnam National University of Agriculture, Ngo Xuan Quang Street, Hanoi 100000, Vietnam; 3 School of Food Science and Nutrition, University of Leeds, Leeds LS2 9JT, UK

**Keywords:** quercetin, chitosan, Captisol^®^, cyclodextrin, nanoparticles, quorum sensing inhibitors, *E. coli* Top 10

## Abstract

The widespread emergence of antibiotic-resistant bacteria has highlighted the urgent need of alternative therapeutic approaches for human and animal health. Targeting virulence factors that are controlled by bacterial quorum sensing (QS), seems a promising approach. The aims of this study were to generate novel nanoparticles (NPs) composed of chitosan (CS), sulfo-butyl-ether-β-cyclodextrin (Captisol^®^) and/or pentasodium tripolyphosphate using ionotropic gelation technique, and to evaluate their potential capacity to arrest QS in bacteria. The resulting NPs were in the size range of 250–400 nm with CS_70/5_ and 330–600 nm with CS_70/20_, had low polydispersity index (<0.25) and highly positive zeta potential ranging from ζ ~+31 to +40 mV. Quercetin, a hydrophobic model flavonoid, could be incorporated proportionally with increasing amounts of Captisol^®^ in the NPs formualtion, without altering significantly its physicochemical properties. Elemental analysis and FTIR studies revealed that Captisol^®^ and quercetin were effectively integrated into the NPs. These NPs were stable in M9 bacterial medium for 7 h at 37 °C. Further, NPs containing Captisol^®^ seem to prolong the release of associated drug. Bioassays against an *E. coli* Top 10 QS biosensor revealed that CS_70/5_ NPs could inhibit QS up to 61.12%, while CS_70/20_ NPs exhibited high antibacterial effects up to 88.32%. These results suggested that the interaction between NPs and the bacterial membrane could enhance either anti-QS or anti-bacterial activities.

## 1. Introduction

The current poor efficacy of antibiotics to treat bacterial disease, due to the increasing widespread emergence of resistance, highlights the urgent need for alternative therapeutic strategies. Rather than focusing on targeting bacteria either by bactericidal or bacteriostatic agents, targeting their virulence and associated factors, seems a more promising alternative approach. Such virulence factors are required for infection (e.g., toxin function and delivery, regulation of virulence expression and bacterial adhesion); they seem to be preserving the endogenous host microbiome and impose less selective pressure on pathogenic bacteria and in theory, decrease resistance [1]. Many bacteria use a cell-cell communication process termed quorum sensing (QS) to communicate, coordinately regulate their gene expression and synchronise their collective social behaviours, such as biofilm formation, bioluminescence and secretion of virulence factors [2,3]. QS involves the production, detection of, and response to extracellular signalling molecules known as autoinducers [4]. QS is not essential for the growth of bacteria though. Thus, its exquisite disruption (known as “quorum quenching”, QQ) offers the possibility to disarm the virulence capacity, rather than killing the bacteria, thus leading to weaken the selective pressure imposed on the pathogens and postpone the evolution of resistance to QQ drugs [5]. Accordingly, this approach appears as a promising strategy for anti-virulence therapy [4]. Nanobiotechnology has opened new possibilities to develop innovative nanomaterial systems with antibacterial and with QQ capacity [6,7]. Ideal QQ therapeutic compounds need not only to exhibit a low toxicity profile, but also suitable pharmacokinetic characteristics for clinical applications. Many potential new molecules reveal promising high QQ efficacy, but they are not considered further due to their low intestinal absorption [8]. As reported by the Biopharmaceutics Classification System, drugs can be classified based on their solubility and permeability. According with this classification, four classes of drugs have been proposed: Class 1 (high solubility, high permeability), Class 2 (low solubility, high permeability), Class 3 (high solubility, low permeability) and Class 4 (low solubility, low permeability) [9]. Except Class 1, compounds in the three remaining groups lack either solubility or permeability, both very important attributes that dictate the bioavailability of drugs through biological membranes. The continuous discovery of many new drug candidates which fail because of low oral bioavailability [10], highlights the need for designing delivery systems capable to overcome these problems [8]. 

Among the drug delivery strategies intended to increase the bioavailability of drugs, the use of polymeric nanocarriers has received significant traction. Chitosan refers to a family of linear, semi-crystalline polysaccharide composed of randomly distributed β (1→4)-linked *N*-acetyl d-glucosamine (acetylated unit) and d-glucosamine (deacetylated unit) and constitute a unique class of biopolymers [11]. Commercially available chitosans produced by chemical methods vary mostly on their degree of acetylation (DA) and molar mass. In addition, using enzymatic and biorefinery approaches, Moerschbacher from our University, has pursued the objective of obtaining a new generation of chitosans with specific non-random PAs [12]. To be termed “chitosan”, the deacetylated chitin should contain at least 60% of d-glucosamine residues [13], which corresponds to a degree of acetylation of 40% (i.e., degree of deacetylation 60%). Chitosan’s DA can vary widely in the range 0 to over ~60%, while the molar mass commonly ranges from ~3 to ~400 kg/mol, depending on the source and preparation method. Chitosan exhibits remarkable unique bioactive properties which include biocompatibility, biodegradability [14,15], bioadhesion, absence of allergenicity and toxicity, anti-hypercholesterolemic [16,17], antibacterial activity [18], along with antifungal [19], mucoadhesive [20,21], analgesic [19] and haemostatic properties [22]. Due to these features, chitosan has been presented as an outstanding candidate for biomedical applications, food industrial applications, cosmetics, and pharmaceutics [16]. The broad spectrum antimicrobial activity of chitosan has been widely applied in food preservation since edible chitosan coatings help to preserve vegetables and meat and fish products [23,24,25] result in reducing the amount of synthetic preservatives. Chitosan has shown effective antibacterial activity in both Gram-positive and Gram-negative bacteria. Greatest growth reduction in gram positive *Bacillus cereus* treated with different Mw chitosan oligomers and polymers ranging from ~3 to ~21 kg/mol was observed with chitosan of Mw ~11 kg/mol and DA ~41% [26]. The same effect has been recorded with gram negative *E. coli* treated with chitosans of different Mw varying from 5 to 91.6 kg/mol where the lower Mw, the better antibacterial activity will be achieved [27]. In another study, the influence of chitosan’s DA on antibacterial activity was conducted, which showed that only chitosans with low DA (4% and 10%) inhibit the growth by 30% after 16 h of fermentation [28]. Despite the existing experimental evidence, the precise mechanisms of the antimicrobial effects of chitosan oligomers and polymers remain elusive to date. Two main mechanisms have been proposed to account for chitosan antibacterial and antifungal activities. The first proposal argues that positively charged chitosan and its derivatives can electrostatically interact with negatively charged groups (exp. phospholipid and proteins) at the surface of the cell membrane, and therefore, alters its permeability leading to cellular leakage. Under this proposal, it has also been suggested that there are hydrophobic interations between chitosan and phopholipid membrane of Gram-negative bacteria [29]. This process would prevent essential materials to enter the cells or/and lead to the leakage of fundamental solutes out of the cell [30]. The second mechanism involves the penetration of water-soluble chitosan into the cytosol and binding to cell DNA (still via protonated amino groups), which would lead to the inhibition of the microbial RNA synthesis and DNA transcription [31]. Chitosan antimicrobial properties might in fact result from a combination of both type of mechanisms [32,33].

In our laboratory, we have been researching on alternative approaches to the indiscriminate use of antibiotics against pathogenic bacteria. In this respect, the development of new chitosan-based nanomaterials aimed to interfere with bacterial quorum sensing, is of particular interest. Quorum sensing enables bacteria respond to differences in cell-density by means of producing and detecting the accumulation of signaling molecules [34]. To this end, we have used an *E. coli* Top 10 biosensor that carries a synthetic genetic device based on the luxR/luxI QS genetic circuitry of *Vibrio fischeri*. This biosensor was originally proposed to detect *N*-(3-oxohexanoyl)-l-homoserine lactone (3OC_6_HSL) [35]. At an adequate concentration, two molecules of 3OC_6_HSL, bind to two molecules of the receptor LuxR, and activate the expression of green fluorescent protein (output), which is under the lux pR promoter from *Vibrio fischeri*. This biosensor achieves a sensitivity towards 3OC_6_HSL of <1 nM [36]. We have successfully developed nanocarriers made of the polysaccharide chitosan (CS) which possess inherent mucoadhesive properties to increase the systemic absorption of drugs [37]. It has been documented that the interaction of CS nanoparticles with the mucus layer facilitates the transport of the associated drug to the underlying epithelium. On the other hand, cyclodextrins (CD), well-known cyclic oligosaccharides, have a hydrophobic central cavity and a hydrophilic outer surface, and hence, they can form inclusion complexes with hydrophobic compounds. The inclusion complexes are known to contribute to enhance the solubility and stability of the drugs [38,39,40,41]. Due to a higher water solubility and a better biocompatibility profile, cyclodextrin derivatives such as sulfobutyl-ether-β-cyclodextrin (SBEβCD), known also under the commercial brand name Captisol^®^, carboxymethyl β-cyclodextrin (CM-β-CD) and hydroxypropyl β-cyclodextrin (HP-β-CD) derivatives, have been used in drug formulation, particularly, for Class 2 and 4 drugs (low solubility and low permeability).

Emerging studies suggest that flavonoid compounds have many significant effects in natural anti-virulence applications [42,43,44,45]. However, many of these compounds suffer from poor water solubility, low chemical stability and little bioavailability [46]. To overcome some of these caveats, it has been proposed that encapsulating in biopolymer-based materials, the activity of anti-virulent agents or anti-microbial could be improved [47,48]. This might be attributed to the increase in their bioavailability, as well as the prolongation in their release with lower doses to overcome the resistances offered by physiological barriers as compared with free agents [49,50]. In this study, we have chosen quercetin as a model flavonoid payload and evaluated the feasibility of its incorporation and delivery using chitosan/SBEβCD-based nanoparticles. Quercetin is a common natural polyphenolic flavonoid compound, found ubiquitously in plants, including food products like onions, many fruits, or in herbs [51,52]. Many reported studies agree that quercetin has a wide range of biological activities including anti-virulence/anti-biofilm formation agent [53], anticancer [54], antioxidant [55] and to reduce the blood pressure in hypertensive subjects [56,57]. However, these activities are somewhat compromised due to the low aqueous solubility and gastrointestinal instability [58]. Quercetin is normally present as a glycoside that is absorbed in the small intestine and converted into glucuronide and sulfate conjugates that render the molecule inactive to allow for its excretion through urine [59]. Most of quercetin occurring in plants as hydrophilic glycosides have limited direct absorption [60]. According to researches on human volunteers, no quercetin was detected in the plasma or urine after oral administration in a dose of 4 g [61]. Furthermore, quercetin has been disappeared immediately from plasma of rodents administered intravenously. This suggested that quercetin could be metabolized speedily and the accumulation in tissues and biological fluids is very negligible [62]. More recent in vivo studies, suggest that quercetin metabolites can also function as carriers that transport quercetin into different tissues, such as vascular, where β-glucuronidase will deconjugate it into the aglycone form which has the most biological effect [57]. It may be that the entrapment of quercetin in chitosan-cyclodextrin nanoparticles might help to improve the solubility, potentiate the biological effects and improve the bioavailability of quercetin in a controlled manner. Chitosan/cyclodextrin nanoparticles have been reported as potential carriers for the oral delivery of small peptides [63] as well as for the gene delivery to the airway epithelium [64]. In the present work, we aimed to design a novel anti-QS formulation that combine the virtues of chitosan and Captisol^®^ nanoparticles in terms of association for quercetin, modulating the release profile and enhancing the anti-QS efficacy using a *E. coli* Top 10 AHL-regulated biosensor.

## 2. Results

### 2.1. Preparation of Unloaded Nanoparticles

**Figure 1:**
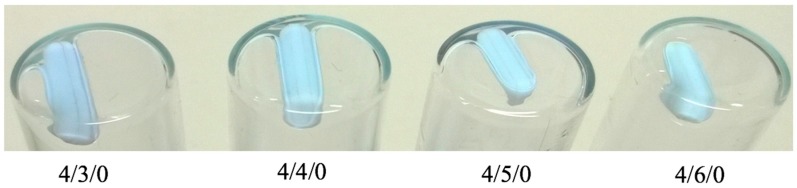
Appearance of CS_70/5_/SBEβCD nanoparticles showing a gel phase at the bottom of the vials that holds the magnetic stirrers.

**Figure 2:**
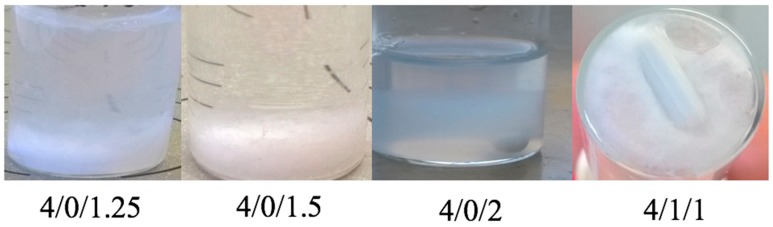
Appearance of various formulations of CS_70/20_/SBEβCD/TPP nanoparticles immediately after being prepared, showing the aggregation phenomenon.

Nanoparticles composed of chitosan, and either SBEβCD or mixtures of SBEβCD/TPP, were obtained via the ionotropic gelation technique [65]. This method is based on the ionic interaction between the positively charged CS and the negatively charged TPP and/or SBEβCD, and the ability of CS to form inter- and intra-molecular linkages with poly-anions thus resulting in the formation of colloidal particles. By contrast with macroscopic gelation, this process occurs in dilute conditions. The process is extremely mild as it only involves the mixture of two aqueous phases at room temperature. Previous studies have reported the use of the neutral hydroxypropyl β-cyclodextrin derivative in association with CS to form nanoparticles [66,67]. In this study we decided to choose a negatively charged cyclodextrin derivative-SBEβCD, which allegedly could be incorporated more effectively into nanoparticles due to stronger ionic interactions with the positively charged CS. Nanoparticles could be prepared either in the presence or absence of TPP by mixing CS with different amounts of SBEβCD (Table 1 and Table 2). The resulting NPs prepared with CS_70/5_ and CS_70/20_ were in the size range of 250–400 and 330–600 nm, PDI 0.03–0.19 and 0.13–0.25, respectively, and invariably high positive zeta potential ranging from +31 to +40 mV. Generally, if the contents of initial anionic charged species (SBEβCD and/or TPP) was too low (e.g., CS/CD/TPP mass ratio 4/1/0 and 4/2/0 in Tables S1 and S2), NPs either did not form or their yields were too low for characterization. On the other hand, too much of initial anionic charged species, resulted in either aggregation or the NPs could not be re-suspended after isolation (Figure 1 and Figure 2). We reasoned that when a fixed amount of CS was used, the amount of cyclodextrin that is adequate for NPs formation varied with the proportion of Captisol^®^ which carries more than six sulfate charges per mol (SBEβCD, D.S. ≈ 6.4), as well as the presence of TPP cross-linker, which is supposed to compete with SBEβCD for the positively charged amino group of CS. If the net charge ratio (^+^/_−_) ranges from 0.75 to 1.25 (near the isoelectric point), precipitation occurred immediately (e.g., CS/CD/TPP mass ratio 4/1.5/1, 4/2/0.75, 4/3/0.5 and 4/4/0.25 in Tables S1 and S2). Around this point, NPs of greater size were obtained (e.g., at CS_70/20_/CD/TPP mass ratio 4/4/0 charge ratio ≈ 1.5). Our results are consistent with previous works [66], that report when the SBEβCD/TPP ratio decreased, the size, zeta potential and production yield of NPs increased (cf. mass ratio 4/1/0.5 vs. 4/2/0.25 in Table 1; and mass ratio 4/1/0.75 vs. 4/2/0.5 in Table 2). The lower zeta potential with increasing SBEβCD amounts in these formulations could be explained by an increased masking of free positively charged amino groups of CS. It also might be noted that TPP incorporated in the formulation helps to increase the production yield.

### 2.2. Preparation and Characterization of Quercetin-Loaded Cyclodextrin-Containing CS Nanoparticles

**Figure 3:**
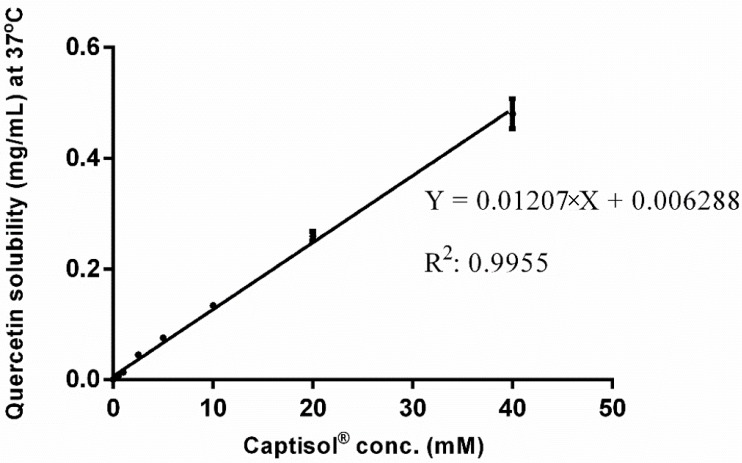
Phase solubility of quercetin in the presence of increasing concentration of SBEβCD.

NPs loaded with quercetin were prepared. To achieve a comprehensive picture of the encapsulation process of this compound in the NPs, phase-solubility studies with increasing SBEβCD concentrations were performed (Figure 3). As expected, quercetin showed a marked increase in their solubility as the SBEβCD concentration increased. In fact, a 325-fold increase in quercetin solubility was achieved using 40 mM SBEβCD solutions [66]. Table 3 and Table 4 show the size, PDI, zeta potential and production yield of quercetin-loaded NPs of CS_70/5_ and CS_70/20_, respectively. In all formulations, positive zeta potential values were detected, suggesting that CS is mainly located on the surface of the particles. The addition of quercetin did not change significantly the physicochemical properties of the NPs, except for the PDI of the CS_70/20_- SBEβCD NP formulations that increased slightly.

**Figure 4:**
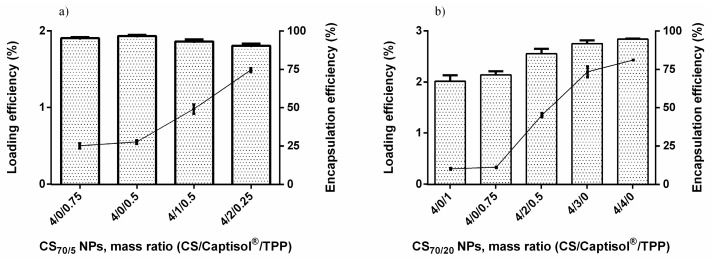
Encapsulation efficiency (grey column) and loading efficiency (black circle) of quercetin in selected loaded nanoparticle formulations of CS_70/5_ (**a**); and CS_70/20_ (**b**).

In previous studies, it has been shown that at least 99% of the maximum drug solubility was already reached in 24 h [66]. This result allowed to reduce drug/SBEβCD incubation time to 24 h for the solutions intended for loaded NP preparation. In the next step, we investigated how the solubilization of quercetin by its inclusion on the CD cavity could facilitate the association of the complexed flavonoid to CS NPs (Figure 4a,b). As the amount of Captisol^®^ increased, the amount of flavonoid-complexed with Captisol^®^, and the final loading of NPs increased too. With CS_70/5_, when compared with the control formulation 4/0/0.75, the loading efficiency (LE) of 4/1/0.5 and 4/2/0.25 increased 1.96- and 2.98-fold, respectively (Figure 4a). With CS_70/20_, when compared with the control formulation 4/0/1, the LE of 4/1/0.75 and 4/2/0.5 increased 2.87- and 4.5-fold, respectively (Figure 4b). Specifically, with NP formulations 4/3/0 and 4/4/0, the LE increase up to 7.33- and 8.1-fold, respectively, when compared with the control formulation, thus suggesting that the LE increased in proportion with the increase of the amount of Captisol^®^. As can observed from Tables S3 and S4, formulations without Captisol^®^, the LE achieved was very low. By contrast, when the amount of TPP decreased, the encapsulation efficiency (EE) and LE were elevated, thus effectively suggesting that TPP might compete with quercetin during the association with CS in these formulations (CS_70/5_ 4/0/1 vs. 4/0/0.75; CS_70/20_ 4/0/0.75 vs. 4/0/0.5).

### 2.3. Elemental Analysis of Selected NPs

**Figure 5:**
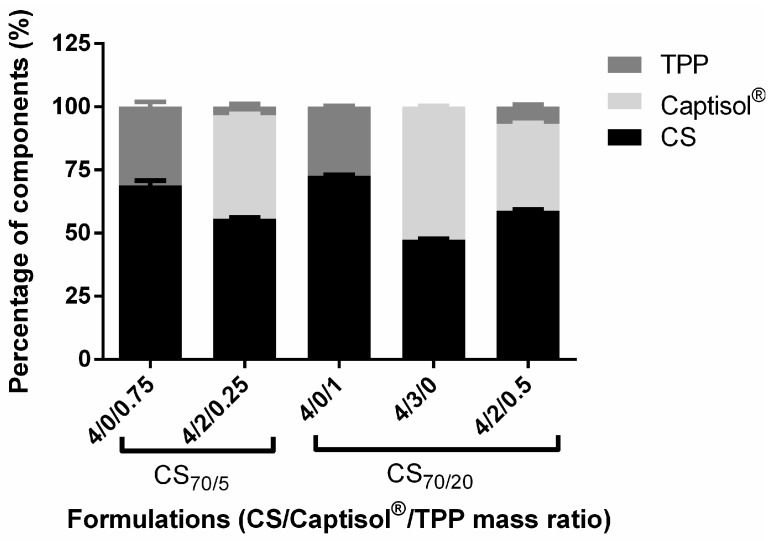
Composition of selected formulations of nanoparticles, as determined by elemental analysis (mean ± S.D., *n* = 3).

Many approaches have been proposed for the quantification of SBEβCD in nanoparticle carrier systems. Most of them rely on colourimetric reactions of the cyclodextrin with an appropriate reagent (e.g., fading of phenolphthalein reaction) [63,68]. These methods are useful, however, the need for either lyophilized of the supernatant of NPs or using the enzymatic reaction at 40 °C for 60 min in 2% starch, have limited their application. In this study, elemental analysis was performed to determine the composition of the different nanoparticle formulations (Figure 5). Using this technique, the composition of the NPs could be determined by comparing the C–N mass ratios (or the C–N–S mass ratios) of CS and SBEβCD with those of the NPs. CS/SBEβCD/TPP (CS_70/5_ 4/0/0.75 and CS_70/20_ 4/0/1) NPs were analyzed and taken as the references for SBEβCD-containing NP formulations. Their compositions were 68.93% CS, 31.07% TPP and 72.73% CS, 27.27% TPP for CS_70/5_ and CS_70/20_ NPs, respectively. These values are close to the expected ones from theoretical ratios at which the materials were incorporated. As expected, the anionic SBEβCD could be incorporated into the NPs with considerable high efficiency: 41.03 and 34.5% (*w*/*w*) of the final composition NPs corresponding to the respective SBEβCD incorporated (CS_70/5_ 4/2/0.25 and CS_70/20_ 4/2/0.5, respectively). Particularly, SBEβCD was effectively entrapped into the CS_70/20_ 4/3/0 NPs, representing up to 52.7% of the total components of the nanoparticles.

### 2.4. Stability Studies 

**Figure 6:**
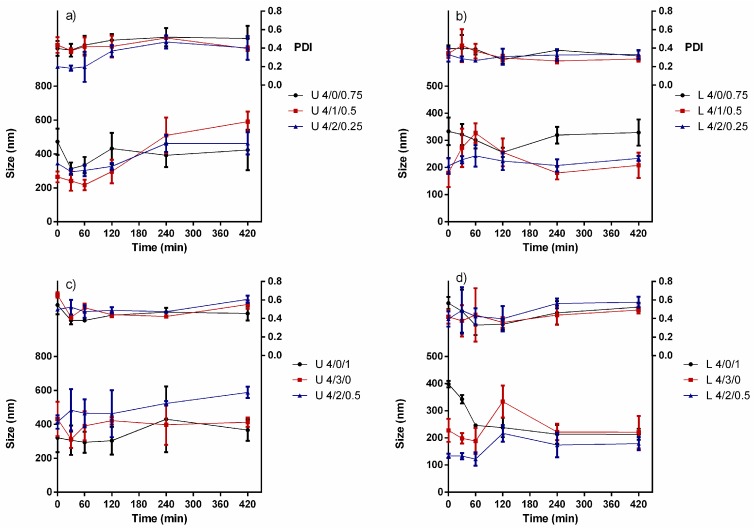
Stability of selected unloaded and quercetin-loaded nanoparticles of CS_70/5_ (**a**,**b**), and CS_70/20_ (**c**,**d**) in M9 medium at 37 °C (mean ± S.D., *n* = 3) at varying mass ratios of CS/SBEβCD/TPP shown in labels (L: loaded NPs; U: unloaded nanoparticles).

As the final intended application of these nanoparticles is their use for anti-QS in gram negative bacteria, we determined their stability in M9 medium (pH 6.8 and 37 °C). The results showed that both loaded and unloaded nanoparticles did not suffer a significant change in their size following incubation for 7 h (Figure 6a–d). The size varied within a small range 300–500 and 200–400 nm for unloaded and quercetin-loaded NPs, respectively. However, upon contact with M9 medium some formulations exhibited a size increase which could be attributed to a swelling effect. There was a slight variation in PDI of these NPs during the first 3 h, after that the PDI tended to stabilize at ~0.5 and ~0.3 for unloaded (Figure 6a,c) and quercetin-loaded (Figure 6b,d) formulations, respectively. Previous studies have also shown the possible role of cyclodexrtins in particle stabilization [66,67,69]. The colloidal stability is very important, since it maximizes the number of NPs covering the surface of bacteria as well as maintaining the inherent surface effect to volume ratio of these NPs.

### 2.5. In Vitro Release of Quercetin 

**Figure 7:**
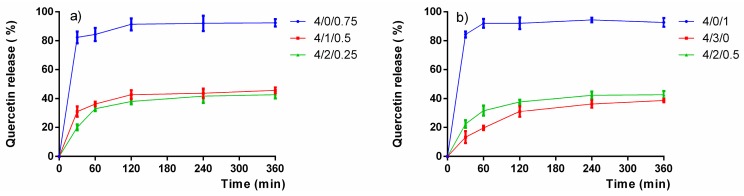
Quercetin in vitro release profile of selected loaded nanoparticles of CS_70/5_ (**a**) and CS_70/20_ (**b**) in M9 medium at 37 °C (mean ± S.D., *n* = 3) at varying mass ratios of CS/SBEβCD/TPP shown in labels.

As can be appreciated from Figure 7, in formulations without Captisol^®^, the encapsulated quercetin was released up to 90% within 60 min. The fast release of quercetin from the nanometric matrix could be explained because of its weak interaction with chitosan. This result was in accordance with previous studies where almost all the payload was released from CS/TPP NPs in 15 min [66,70]. In contrast, nanoformulations containing Captisol^®^ seem to prolong the release of the loaded-drug. The different composition of these NPs regarding different amounts of TPP and Captisol^®^ has a negligible influence on the release profile of quercetin when around 40% of quercetin was released after 6 h incubated in M9 medium at 37 °C. The slow release of quercetin in these formulations could be understood as the expected consequence of the inclusion complexes formed by the hydrophobic cavity of Captisol^®^ and quercetin. The strong interaction between drug and Captisol^®^ might have an impact in controlling the drug release. Previous studies have reported the ability of Captisol^®^ to form inclusion complexes with auto-inducers, especially with AHL with acyl tail from C_4_ to C_8_ [71,72,73,74], hence we speculated that the drug release rate might be increased significantly when AHL is added to the bacterial medium leading to the competition of AHL and quercetin to occupy the cavity of Captisol^®^. The simultaneously burst release of loaded-drugs (vancomycin and hamamelitannin) within 1 h and the uptake auto-inducers (either C_6_HSL or 3-oxo-C_12_HSL) was reported elsewhere [75].

### 2.6. FTIR Analysis of Selected NPs

**Figure 8:**
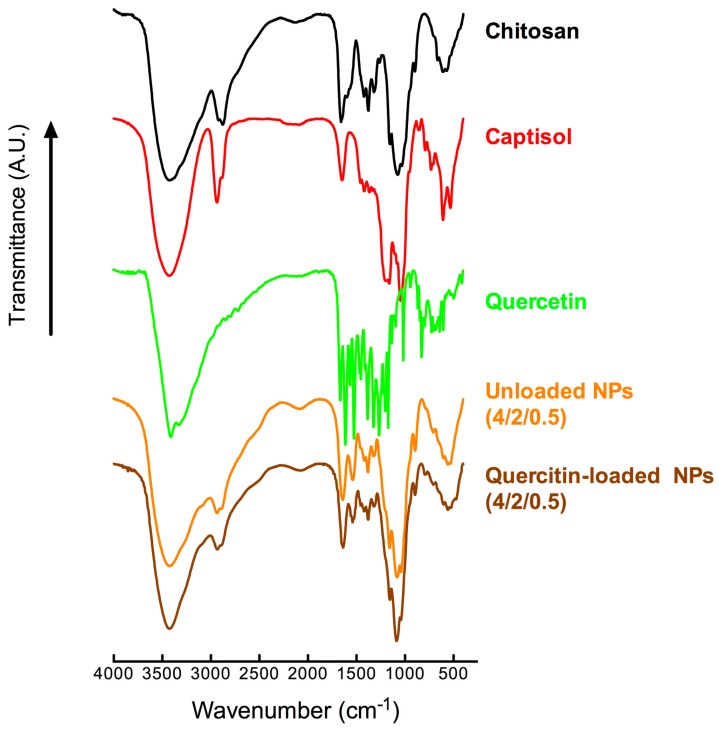
FTIR transmission spectra of free chitosan, Captisol^®^, quercetin, unloaded and quercetin-loaded CS/SBEβCD/TPP 4/2/0.5 nanoparticles.

Fourier transform infrared spectroscopy (FTIR) analyses were performed on freeze-dried samples of selected loaded NPs to identify the infrared absorption peaks of quercetin, chitosan, Captisol^®^, unloaded, quercetin-loaded NPs and to investigate a possible reaction between quercetin and NPs (Figure 8).

The FTIR spectrum of free quercetin displayed bands and typical molecular peaks of its structure such as: 1381 cm^−1^ (C–OH), 1610 cm^−1^ (C=C), 1262 cm^−1^ (C–O–C), 1662 cm^−1^ (C=O), and 3408 cm^−1^ (O–H stretch). CS_70/20_’s FTIR spectrum showed its characteristic bands with peaks at 1657 cm^−1^ and 1599 cm^−1^ attributed to NH-bending units of glucosamine. The absorption bands at 1033 cm^−1^ (C−O–C) and 1075 cm^−1^ (skeletal vibration involving the C–O stretching) are attributed to its regular saccharide structure. In loaded NPs 4/2/0.5, the O–H stretch at 3425 cm^−1^ attributed to O–H stretch of both chitosan and Captisol^®^ remained, while the O–H stretch at 3408 cm^−1^ of free quercetin disappeared. The disappearances of both aromatic bending and stretching (1610 cm^−1^, 1662 cm^−1^) and the peaks between 1200 and 1300 cm^−1^, especially the (C–O–C) peak at 1262 cm^−1^, together with the appearance of new glycosidic linkage peak at 1040 cm^−1^ (shifted from 1042.87 cm^−1^ of Captisol^®^) indicated that quercetin might be entrapped inside the cavity of Captisol^®^ rather than present on the surface of nanoparticles. A new peak appeared centered at 794.5 cm^−1^ (attributed to a peak at 795.92 cm^−1^ of free quercetin) in loaded 4/2/0.5 NPs when compared with unloaded formulation indicated that quercetin was efficiently associated in the NPs. The disappearance of typical peaks of quercetin after nanoencapsulation has been reported elsewhere [55,76]. FTIR results have confirmed the conjugation between quercetin and the NPs matrix.

### 2.7. Bioassay against E. coli Top 10 of Selected NPs

**Figure 9:**
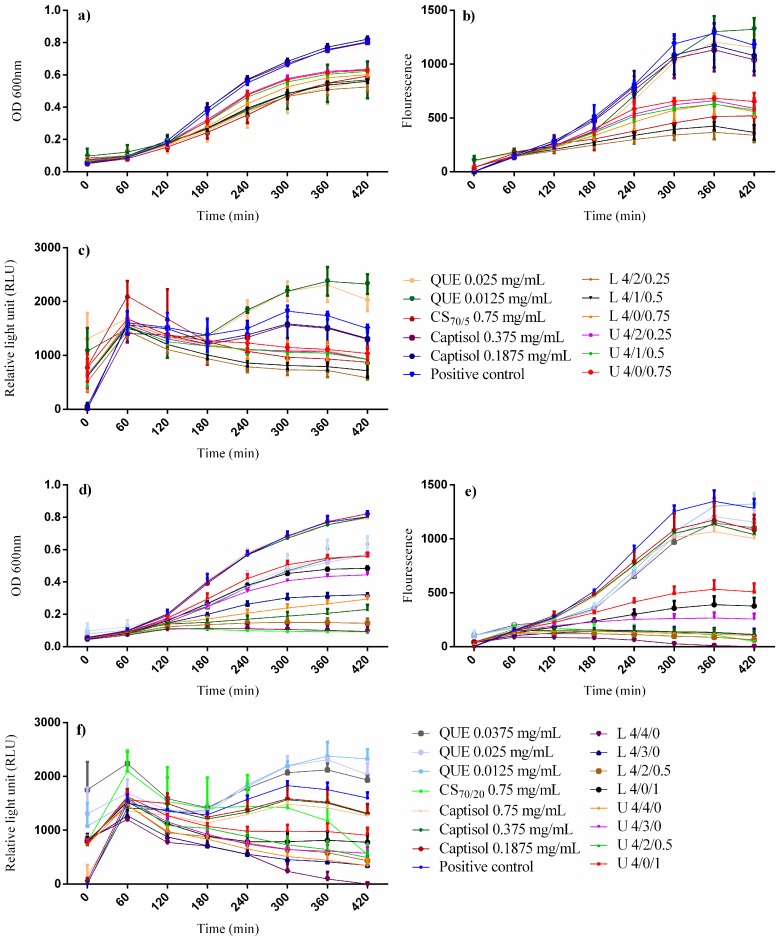

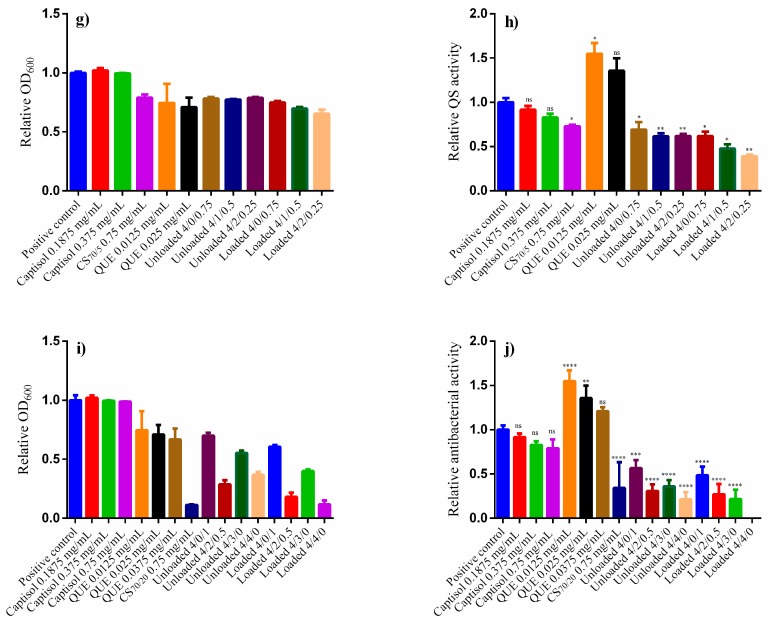
Influence of different treatments on the evolution of bacterial growth (OD_600_), florescence intensity and relative light unit (FL/OD) of CS_70/5_ NPs (**a**–**c**); of CS_70/20_ NPs (**d**–**f**), respectively. Effect of different formulations on relative bacterial growth (OD_600_) and relative QS activity of CS_70/5_ NPs (**g**,**h**); of CS_70/20_ NPs (**i**,**j**), respectively. (L: loaded NPs, U: unloaded NPs, QUE: quercetin; values represent mean ± SD, *n* = 3 with eight technical replicates, statistical significance after ANOVA multiple comparison analysis with respect to the positive control: * *p* < 0.05, ** *p* < 0.01, *** *p* < 0.001 and **** *p* < 0.0001).

We have investigated the influences of free quercetin, chitosan, Captisol^®^, unloaded and quercetin-loaded nanoparticles at different concentrations to the responses of AHL-regulated biosensor strain, *E. coli* Top 10, regarding the evolution of the fluorescence intensity and the bacterial growth (proportional to OD_600_). The ratio between fluorescence intensity and OD_600_ was also calculated and be defined as relative light unit (RLU). To establishing quantitative comparisons, we have selected measurement of the last RLU and OD_600_ (i.e., endpoint measurement after 7 h when the growth rate is assumed to enter the stationary phase). The QS in the positive control was set as 100%, and the relative QS of a given treatment is defined as the ratio of its RLU at 7 h with respect to that of the control. Therefore, theoretically, if the relative QS values are equal to one, it means that the evaluated compounds do not have any anti-QS effect. In turn, relative QS values lower than one, are diagnostic of QS inhibition as the OD_600_ does not decrease. The recorded results are shown in Figure 9. There was no inhibition effect to bacterial growth at different concentrations of Captisol^®^ (Figure 9a,d) namely, 0.1875, 0.375 and 0.75 mg/mL, which are equivalent to the amount of Captisol^®^ in 4/1/-, 4/2/-, 4/4/-NP formulations, respectively, thus suggesting that Captisol^®^ is non-toxic to bacteria.

However, GFP has reduced significantly in a dose-dependent manner in a range of Captisol^®^ from 0.1875 to 0.75 mg/mL (Figure 9b,e). Altogether, increasing amounts of free form Captisol^®^ decreased proportionally relative QS activity from 8.43% to 20.86% (Figure 9h,j). Since the final concentrations of quercetin of loaded-NPs in the bioassays ranging from 0.0028 mg/mL (lowest in CS_70/20_ 4/0/1) to 0.0373 mg/mL (highest in CS_70/20_ 4/4/0 NPs), quercetin existing in free form at three different concentrations namely 0.0125, 0.025 and 0.0375 mg/mL was also tested. CS_70/5_ and CS_70/20_ at the same final concentration in the bioassay (0.75 mg/mL) were also tested. Interestingly, quercetin existing in free form exerted inhibition effect to bacterial growth as evidenced in Figure 9a,d, but the reduction of GFP expression is negligible and the differences between treatments are not very clear. Thus, the free form quercetin exhibited slightly anti-bacterial effect rather than anti-QS effect (Figure 9h,j). In CS_70/5_ NPs, chitosan in free form and unloaded NPs showed negligible inhibition on bacterial growth around 20% (Figure 9a,g). When compared with unloaded NPs, quercetin-loaded ones revealed a minor decrease on bacterial growth that might attributed to the final amount of quercetin-loaded in these formulations, ranging from 0.0046 mg/mL in 4/0/0.75 to 0.0178 mg/mL in 4/2/0.25 (Table S3). Of note, when GFP intensities of loaded-NPs are the lowest (Figure 9b), the bacterial growth seems to be affected very little by CS_70/5_ NPs, thus displaying an anti-QS effect. In fact, when applied in free form, the highest anti-QS effect observed for Captisol^®^ and CS_70/5_ were 20.86% and 27.0%, respectively (Figure 9h).

The anti-QS effect of CS_70/5_ NPs both quercetin-loaded and unloaded increased significantly when compared with the single components. Interestingly, unloaded 4/2/0.25 containing a double amount of Captisol^®^ exhibited equivalent anti-QS effect when compared with unloaded 4/1/0.5. Higher surface charge of unloaded 4/1/0.5 than unloaded 4/2/0.25 nanoparticles (cf. ζ ~+38 vs. +36.3 mV, respectively) could be an explanation for this phenomenon since electrostatic interaction between oppositely surface charged of NPs and bacteria favor anti-QS efficiency. It should be noted that in each formulation, loaded NPs showed stronger anti-QS effect than the unloaded ones, thus suggesting that quercetin might act synergistically with chitosan and Captisol^®^ in increasing the anti-QS effect of these NPs system. As we expected, the highest anti-QS effect, up to 62%, was observed in loaded 4/2/0.25 formulation comprising the greatest amount of Captisol^®^, thus the concomitant greatest amount of associated quercetin.

With CS_70/20_ NPs, free form of CS_70/20_ exhibited significantly inhibitory effect to bacterial growth when 88.67% OD_600_ reduction was observed in this treatment (Figure 9i). The lower OD_600_ reduction observed in unloaded NPs (from 30.1% in unloaded 4/0/1 to 71.33% in unloaded 4/2/0.5) revealed that upon nanoencapsulation, the toxicity of CS_70/20_ was significantly reduced (Figure 9d,i). The same trends were observed when in all formulations loaded NPs showed stronger inhibition effect to bacterial growth (from 39.48% in loaded 4/0/1 to 88.32% in loaded 4/4/0 formulations) than the unloaded ones (Figure 9d,i). Since almost both unloaded and loaded formulations of CS_70/20_ NPs, caused strong reduction in OD_600_ (up to 88.32% in loaded 4/4/0 NPs), these NPs exhibited antibacterial effect rather than anti-QS effect. The reduction in GFP intensity might stem from the death bacteria that cannot generate GFP, rather than the inhibition to survival bacteria expressing GFP. The antibacterial effect in these NPs could be divided into two main groups. The first group which caused slight ^[+]^/_[−]_ antibacterial effect including unloaded NPs (except for 4/2/0.5 and 4/4/0 formulations), loaded 4/0/1 and loaded 4/3/0. The second group which caused strongly antibacterial effect including both unloaded and loaded of 4/2/0.5 and 4/4/0 formulations, free CS_70/20_ (0.75 mg/mL). In general, formulations containing Captisol^®^ inhibited bacterial growth stronger when compared with the control (formulation 4/0/1 both unloaded and loaded NPs). In unloaded NPs, except for 4/2/0.5 formulation, the antibacterial effect increased proportionally with the amount of Captisol^®^ (4/3/0 vs. 4/4/0 NPs) highlighting the crucial role of Captisol^®^’s cavity in inhibiting bacterial growth and florescence as well. The stronger effect observed in unloaded 4/2/0.5 when compared with unloaded 4/3/0 and unloaded 4/4/0 could be explained by the higher positive surface charged of this NPs formulation (ζ~ +40 mV in 4/2/0.5 vs. ~+32 mV in 4/3/0 and 4/4/0 (Table 2). With the loaded NPs, the higher amount of quercetin loaded in the NPs matrix, the higher antibacterial activities were observed with 4/3/0 and 4/4/0 formulations. The higher activity of loaded 4/2/0.5 NPs than loaded 4/3/0 NPs suggesting the synergistic effect between highly positive surface charged of 4/2/0.5 NPs with the natural Captisol^®^’s cavity and the antibacterial effect of quercetin as well. It should be noted that free quercetin and 4/0/1 NPs caused slightly antibacterial effect on bacteria. However, both unloaded and loaded 4/0/1 NPs could reduce the GPF expression more efficiently when compared with free quercetin of different concentrations. Quercetin-loaded NPs showed less toxicity to bacterial than free chitosan, but stronger reduction of GFP were observed with loaded 4/2/0.5 and loaded 4/4/0 NPs suggesting that encapsulation process could reduce the toxicity of NP’s component as well as enhance GFP inhibitory activities of these NPs. The lowest florescence intensity observed in loaded 4/4/0 formulation suggests that at this concentration (drug releases up to ~40% from the nanoparticle matrix after 6 h, was equivalent to 0.0298 mg/mL) might be too high and caused the toxic for this biosensor bacteria.

## 3. Discussion

CS/SBEβCD nanoparticles were prepared by ionic gelation either in the presence or absence of TPP. Nanosystems were formed by the combination of the electrostatic interaction between CS and SBEβCD, which are oppositely charged, and the ability of CS to experience a liquid–gel conversion due to its ionic interaction with TPP. The initial experiments were aimed at screening the best NPs formulations using a derivative of β-CD with degree of substitution of 6.4 (SBEβCD) and two kinds of chitosan namely CS_70/5_ and CS_70/20_. As can be seen from the results (Table 1, Table 2, Table 3 and Table 4; Figure 1 and Figure 2), the incorporation capacity of CS could be determined by their degree of acetylation (DA), which are corresponding to their positive charge. CS_70/20_ with DA of 21% has shown the higher ability to incorporate with negatively charged of SBEβCD or mixture of SBEβCD/TPP when compared with CS_70/5_ with DA of 32.4%. The best mass ratio (CS/SBEβCD/TPP) formulations of CS_70/5_ were 4/1/0.5 and 4/2/0.25 whereas those comprising CS_70/20_ were 4/1/0.75 and 4/2/0.5. Indeed, the ^[+]^/_[−]_ charge ratio plays an important role in the formation of NPs. In this study, we have shown that around the stoichiometric point where charge ratio ^[+]^/_[−]_ ≈ 1, NPs could not form and aggregation occurred immediately. This important result allowed shortening the screening processes for the best formulations. Charge ratio can also influence the physicochemical properties of the NPs in terms of size, PDI, zeta potential and production yield. The resulting NPs were in the size range of 250–400 nm with CS_70/5_ and 330–600 nm with CS_70/20_, low polydispersity index (<0.25) and always exhibit high zeta potential (ranging from ζ +31 to +40 mV), thus suggesting that CS is mainly located on the surface of the particles. It could be noted that production yield of formulations containing TPP increased significantly. Quercetin, a poorly soluble flavonoid, was chosen for testing the ability to load hydrophobic drug of the best NPs formulations in previous part. Quercetin-loaded NPs were characterized in terms of size, PDI, zeta potential and production yield. The results show that the addition of quercetin did not alter significantly the physicochemical properties of the NPs, suggesting that quercetin was fully entrapped in the cavity of cyclodextrin. The noncovalent inclusion complexes formed by Captisol^®^’s cavity and guest molecules both in solution and the solid state can lead to alter the physical, chemical and biological properties of guest molecules. The inclusion complexes in which guest molecule was surrounded by hydrophobic environment of Captisol^®^’s cavity is ideal for delivering low solubility drug. Solid inclusion complexes between quercetin and Captisol^®^ have also been studies before in order to enhance the solubility, dissolution rate, as well as improve significantly anti-cancer activity at lower quercetin concentration [77]. Quercetin was released sustainable but higher antioxidant activity and photostability was obtained upon inclusion complexed with β cyclodextrin [78]. The toxicity of quercetin has also been demonstrated to be reduced upon complexing with hydroxypropyl β-cyclodextrin elsewhere [79]. Results from Table 3 and Table 4 indicate that Captisol^®^ could facilitate the association of complexed drug into the CS NPs. The association efficiency in all formulations containing Captisol^®^ was higher than 85% and the loading efficiency increased linearly with Captisol^®^ amount. When compared with the controls (4/0/0.75 in CS_70/5_ NPs and 4/0/1 in CS_70/20_ NPs), the LE of the best formulation increased up to 2.98 and 8.1 times, respectively. Interestingly, the LE of the controls (without Captisol^®^) were negligible and when the amount of TPP decreased, the AE and LE increase suggesting that TPP might compete with quercetin in association with CS in these formulations.

Elemental analysis was performed to identify the compositions of selected NP formulations. The values obtained by this technique are close to the theoretical mass ratios at which the materials were incorporated. As expected, Captisol^®^ was effectively incorporated into the NPs and representing up to 52.7% of the total mass in CS_70/20_ 4/3/0 NPs. The preparation of nanoparticles containing more than 50% mass of SBEβCD is very crucial since SBEβCD is low toxicity and possess special features in terms of enhancing permeability and protecting drug molecules [80]. The strong interaction between the SBEβCD and CS is afforded by the presence of negatively charged sulfate groups in the SBEβCD that ionically interacts with the positively charged CS molecules.

FTIR results indicated that quercetin was effectively entrapped inside the cavity of Captisol^®^. This result is very important since quercetin will be released gradually from the nanosytems that was driven by the exchange between quercetin and autoinducer (3OC_6_HSL) to occupy the cavity of Captisol^®^. The gradualy release has allowed bacteria to have enough time to adapt to the drug and therefore, help to reduce the toxicity of the nanosystems as well as prolong their anti-QS effect.

Bioassays against *E. coli* Top10 biosensor have been carried out to evaluate the bioactivities of NPs derived from two kinds of chitosan with different DA. CS_70/5_ NPs exhibited highly anti-QS effect while CS_70/20_ NPs showed strongly antibacterial effect. This suggests that CS’s DA is a crucial factor that determine the pathway in which NPs might interfere with bacteria. In fact, both anti-QS and anti-bacterial activities increased significantly upon nanoencapsulation, thus highlighting the benefit of unique physicochemical properties and high surface area to volume ratio of NPs that facilitate their attachment to bacteria’s membrane and enhance the bioactivities effect of the systems as well as minoring their toxicity. As evidenced in Figure 9, the best anti-QS and anti-bacterial effects were attained in loaded NPs, suggesting that the synergistic effect of chitosan, Captisol^®^ and quercetin will optimize the bioactivities of nanosystems. Formulations containing Captisol^®^ (both loaded and unloaded) showed higher either anti QS or antibacterial effects than the control without Captisol^®^ (4/0/0.75 and 4/0/1, respectively). This suggests that the exchange between the release of quercetin outside Captisol^®^’s cavity and the simultaneously uptake of 3OC_6_HSL inside this cavity could be the reason of enhancing bioactivities of these NPs. The uptake of autoinducer inside the cavity making autoinducer cannot reach adequate threshold to activate the fully QS in *E. coli* Top10 biosensor. Our result is in accordance with previous works [73,74,81] that suggested autoinducers, especially AHLs possessing an acyl chain from C_4_ to C_8_ (in our case is 3-oxo-C_6_-HSL), could be trapped inside the cavity of Captisol^®^, thus leading to the reduction in QS activity. Quercetin at concentration of 16 μg/mL has been reported sofar as an effective inhibitor of QS, biofilm formation and QS-regulated virulence factors in *P. aeruginosa* PAO1 [82]. The two main QS systems in *P. aeruginosa* PAO1 are lasI/R and rhlI/R in which lasI and rhlI are involved in autoinducer synthesis, while lasR and rhlR served as receptors. *E. coli* Top 10, biosensor used in our studies, possesses a cassette luxR transformed from *Vibrio fischeri* that can only respond to 3OC_6_HSL but cannot produce autoinducer due to lack of luxI cassette. Since QS circuits in *P. aeruginosa* PAO1 and *E. coli* Top 10 respond to different type of autoinducers and quercetin exerted anti-QS effect on both systems, we can excluded that quercetin compete for the binding site of the involved receptors with the cognate autoinducers and different mechanisms have been hypothesized. One possibility could be that quercetin inhibited autoinducer synthase enzymes that could be eliminated from our studies, as *E. coli* Top 10 does not synthesize autoinducer. Also, quercetin can bind to different domains of LuxR receptor (except for binding site) that would affect the binding affinity of luxR to luxI-DNA. An alternative explanation is that quercetin might be accumulated rapidly at the lipidic membrane of bacteria leading to block the diffusion of AHL to the cytosol [83]. The last interpretation seems to be the most plausible one, as it can answer why in free form quercetin exert highly toxic to bacterial growth in a dose dependent manner while CS_70/5_-loaded NPs do not. In one hand, quercetin was released in a sustained and controlled manner from the NPs, permitting bacteria to have enough time to adapt to as well as metabolise this drug. On other hand, the aggregation effect due to the highly positive charged of these NPs could enhance the anti-QS of the system since they can deliver their payloads locally at the bacterial cell wall, maintain the lower dose during the timespan of the experiment and hence prolong the anti-QS effect of the nanosystems. In addition, our previous study has shown that blank nanocapsules could 100% bind to *E. coli* Top 10 at their low concentration below the optimal “stoichiometric” nanocapsule/bacterium binding point [84]. However, the precise mechanisms underlying these effects remain to be fully elucidated, and the results need to be confirmed in an in vivo experiment. As a natural QS inhibitor, quercetin has several advantages. Firstly, quercetin is low in cost and abundant in nature. Secondly, as reported by available literatures, quercetin would not have any adverse health effect on human following the oral administration at doses up to 1000 mg per day for up to 12 weeks [85]. Thirdly, the anti-QS effect observed for quercetin is at very low concentrations, compared with most of plant extracts and substances before [86,87]. To this end, nanoencapsulation could help to enhance both anti-QS and anti-bacterial effect of the nanosystems. Chitosan’s DA plays an important aspect in determining their pathways to interfere with bacteria.

## 4. Materials and Methods 

### 4.1. Materials

Chitosan samples were of high purity research grade from Heppe Medical Chitosan GmbH (Halle/Saale, Germany), namely sample Code HMC 70/5 (Batch No. 212-170614-01; degree of acetylation 32.4% which corresponds to a degree of deacetylation of 67.6%, as determined by ^1^H-NMR; molar mass 17.6 Kg·mol^−1^ as determined by intrinsic viscosity in 0.3 M acetic acid/0.2 M sodium acetate at 25 °C) and sample Code 70/20 (Batch No. 212-100715-03; degree of acetylation of 21% which corresponds to a degree of deacetylation of 79% and molar mass 78.6 Kg·mol^−1^, determined by the same methods as the HMC 70/5 sample). Both chitosan samples used in this study were originated from crab shell waste by thermoalkaline deacetylation. Sulphobutyl ether-β-cyclodextrin sodium salt (SBEβCD, Mw = 2163, substitution degree ≈ 6.4) was a kind gift from CyDex, Inc. (Lenexa, KS, USA). Pentasodium tripolyphosphate (TPP), Quercetin (Mw = 302.24, Log P: 2.16) and 3OC_6_HSL were analytical grade and were all purchased from Sigma-Aldrich GmbH (Steinheim, Germany). Ultrapure Milli-Q water was used throughout.

### 4.2. Methods

#### 4.2.1. Phase-Solubility Studies

To get insight the kinetics and dynamics of quercetin’s solubility, phase-solubility studies were performed by adding an excess of the drug to 5 mL solution containing increasing amounts of SBEβCD (from 0 to 40 mM) in sealed glass containers stirred at 37 °C until equilibrium (after 3 days). The suspension was then filtered (pore size 0.45 µm), and quercetin concentration was identified spectrophotometrically (λ = 374 nm) (Jasco V-630 spectrophotometer, Labor und Datentechnik, 64319 Pfungstadt, Germany). In keeping with Higuchi and Connors [88], the apparent 1:1 stability constants were calculated from the straight-line portion of the phase solubility diagrams.

#### 4.2.2. Preparation of Nanoparticles (NPs)

NPs composed of CS and SBEβCD or mixtures of SBEβCD/TPP were obtained via the ionotropic gelation technique [65] with slight modifications. The methods are detailed briefly below:

(a) NPs without CD: were spontaneously formed at room temperature upon addition of 1 mL of TPP aqueous solution (0.15% *w*/*v*, polyanionic phase) to 3 mL of the CS solution (0.20% *w*/*v*, pH 4.95, polycationic phase) under stirring (850 rpm, 10 min). The solution was then kept stable for at least 50 min to allow the complete stabilization of the system.

(b) NPs containing CD: The volumes of the two phases were always the same as well as for NPs without CD. CS/SBEβCD/TPP NPs were prepared by mixing the CS solution (0.2% *w*/*v*) with a polyanionic phase containing SBEβCD (0.15–0.9% *w*/*v*) or both SBEβCD and TPP (0.075–0.3% *w*/*v*).

(c) Preparation of quercetin-loaded nanoparticles: For the association of quercetin into the NPs system, an excess of quercetin was incubated under magnetic stirring (500 rpm, 24 h) with either water solution containing different amounts of SBEβCD (from 3.0 to 6.0 mg/mL) or CS solution (0.2% *w*/*v*, pH 4.95). After incubation, the drug suspensions were filtered through 0.45 µm membrane and the resulting solutions was identified spectrophotometrically for quercetin content. This inclusion-complexed solution was then used for NP formation by the ionotropic gelation technique as described above.

The resulting NPs were isolated by ultracentrifugation on a glycerol bed (10,000× *g*, 40 min, 15 °C; Mikro 220 R, Hettich GmbH & Co. KG, Tuttlingen, Germany). Supernatants were collected for determination of the amount of unbound quercetin. NPs were then re-suspended in 100 µL NaCl 85 mM. Glycerol was used to enhance the re-suspend ability of centrifuged nanoparticles. 

The production yield of the nanoparticles was obtained by centrifuging fixed volumes of the freshly prepared nanoparticles suspensions (16,000× *g*, 40 min, room temperature) without glycerol bed. The supernatants were then discarded, and the pellets were lyophilized at −50 °C until constant weight (after 2 days). The production yield was calculated by comparing the actual weight with the theoretical weight of the total components of nanoparticles.

#### 4.2.3. Physicochemical Characterization of Nanoparticles

The Z-average particle size (hydrodynamic diameter) and size distribution of the NPs were determined by dynamic light scattering with non-invasive back scattering (DLS-NIBS) at 25 °C detected at an angle of 173° fitted with a red laser light output (λ = 632.8 nm) using a Malvern Zetasizer Nano ZS instrument (ZEN3600, Malvern Instruments Ltd., Malvern, UK). The ζ-potential was measured by phase analysis light scattering and mixed laser Doppler velocimetry (M3-PALS) at 25 °C. The samples were diluted 1:20 in 1 mM KCl before measurement.

#### 4.2.4. Elemental Analysis of the NPs

Unloaded nanoparticles were prepared as described above, without using glycerol bed during centrifugation, and finally lyophilized (Telstar Cryodos, Terrassa, Spain). Elemental analysis of the starting materials (i.e., pure CS and pure SBEβCD) and the lyophilized nanoparticles was analyzed by Elemental Analyzer Telstar (Telstar Cryodos freeze-drier, Telstar Industrial SL, Terrassa, Spain). For all samples, the elemental composition of C, H and N was determined. For the SBEβCD (pure component) and CS/SBEβCD NPs, the corresponding composition of S was also determined. The CS content of the NPs sample was analyzed by comparing the N content between the samples and pure CS. For SBEβCD content determination, the C content of the samples arising from CS was calculated and subtracted from the total C amount. The remaining amount of C was used to calculate the amount of SBEβCD by comparing that C amount with that of pure SBEβCD. In the case of the CS/SBEβCD/TPP NPs, the S content was also used for SBEβCD quantification. Hence, the reported data represent an average of the two results. The remaining fraction of the NPs composition was attributed to TPP.

#### 4.2.5. Loading and Association Efficiency of Nanoparticles

The association efficiencies of the nanoparticle formulations were determined after isolation of nanoparticles by centrifugation as described in Section 4.2.2. Supernatants were collected for determination of the amount of unbound quercetin using spectrophotometry method. The loading efficiency (LE%) and the association efficiency (AE%) of quercetin were calculated according with Equations (1) and (2), respectively, namely:

(1) $$\mathrm{Loading}\;\mathrm{efficiency}\;\left(\%\right)=\frac{{\mathrm{Total}}_{\mathrm{drug}-\mathrm{mass}}-{\mathrm{Free}}_{\mathrm{drug}-\mathrm{mass}}}{{\mathrm{Nanoparticles}}_{\mathrm{mass}}}\times 100$$

(2) $$\mathrm{Association}\;\mathrm{efficiency}\;\left(\%\right)=\frac{\left[{\mathrm{Total}}_{\mathrm{Drug}}\right]-\left[{\mathrm{Free}}_{\mathrm{Drug}}\right]}{\left[{\mathrm{Total}}_{\mathrm{Drug}}\right]}\times 100$$

#### 4.2.6. Stability Study in M9 Medium

Stability study has been conducted following the protocol used in our previous studies [89]. Briefly, selected nanoparticle formulations were prepared and centrifuged in the presence of glycerol bed. Unloaded-nanoparticles and quercetin-loaded nanoparticles were tested for their stability in M9 medium in terms of the change in size and the polydispersity index (PDI) of nanoparticles and possible precipitations. Nanoparticles were incubated in M9 medium at 37 °C with agitation of 100 rpm. The size distribution of the nanoparticles and PDI were measured by photon correlation spectroscopy at different time points of 0, 30, 60, 120, 240 and 420 min. Each experiment was performed in triplicates.

#### 4.2.7. In Vitro Release Studies

It was done according to method developed by Kaiser et al., with slightly modifications [49]. Briefly, quercetin-loaded NPs were isolated and re-suspended in NaCl 85 mM. The release studies were performed by incubating 800 µL Quercetin-loaded NPs suspension in 25 mL of M9 medium at 37 °C and then, stirred at 100 rpm. At appropriate time points, the samples were withdrawn, replaced by fresh M9 medium and centrifuged at 16,000× *g* for 30 min. The drug released from the NPs, present in the supernatant, was determined by UV/Vis spectrophotometry at 374 nm and was calculated by interpolation using a calibration curve.

#### 4.2.8. FTIR Spectroscopy Studies

The spectra of Fourier transform infrared spectroscopy (FTIR) was used to analyze molecular bonding formation between QUE and chitosan nanoparticles. The FTIR spectra of pure QUE and QUE-loaded chitosan nanoparticles were recorded using Perkin Elmer Nicolet 520 spectrophotometer (Perkin Elmer, Boston, MA, USA). The lyophilized samples were ground with spectroscopic grade potassium bromide (KBr) powder and then, pressed into 1 mm pellet for FTIR measurement in the range of 450–4000 cm^−1^ with 4 cm^−1^ resolution, using 16 scans. All samples were analyzed and recorded in triplicates.

#### 4.2.9. QS Inhibition Studies

(a) *E. coli* Top 10 Biosensor Strain

The bacterial strain used for all experiments was a fluorescence biosensor constructed from an *E. coli* Top 10 (Invitrogen, Life Technologies Co., Paisley, UK), which had been transformed chemically by Celina Vila of our laboratory to contain the standard biological part BioBrick_T9002 on the plasmid BBa pSB1A3 (http://partsregistry.org/Part:BBa_T9002), kindly donated by Prof. Anderson’s lab (UC Berkeley, Berkeley, CA, USA). The sequence BBa T9002, comprised the *luxR* gene, coding for the transcriptional factor LuxR, under the control of the pTetR promoter, being expressed in a constitutive manner. Upon external addition of 3OC_6_HSL, the dimerization of two monomeric species of LuxR, each bound to one AHL molecule, drives to activation of *gfp* expression through binding of the LuxR-AHL dimerized complex to the lux pR promoter from *Vibrio fischeri*, and initiates the production of green fluorescent protein when 3OC_6_HSL is added. This strain has been used in previous studies in our laboratory [36,84].

(b) *E. coli* Top 10 Biosensor Assay

The bacterial strain was cultivated in Luria-Bertani (LB) medium supplemented with 200 µg/mL ampicillin for 18 h at 37 °C, shaking at 100 rpm and then was stored at −80 °C in 30% sterile glycerol for future use. Before the biosensor assay, the bacteria working solution was prepared by cultivating 40 µL of bacteria stored in cryo-tube at −80 °C into 20 mL M9 medium plus 20 µL of ampicillin (200 µg/mL), under incubation at 37 °C, 100 rpm, until the OD_600_ reached 0.04~0.07 (~4 h). 3OC_6_HSL was dissolved in acetonitrile to a stock concentration of 100 mM and stored at −20 °C. Five µL aliquot of the 100 mM 3OC_6_HSL stock solution was diluted with sterile milli-Q water to a working concentration of 10 nM. QS inhibition activity was tested in 96-well microplate, in which 10 µL of 10 nM 3OC_6_HSL, 10 µL of the treatment nanoparticles formulations and 180 µL aliquots of the bacterial culture of OD_600_ 0.04~0.07 were added. Two kinds of blank were set up. Blank 1 (OD blank) contained 180 µL of M9 medium and 20 µL of milli-Q water. Blank 2 (flourescence blank) contained 180 µL of bacterial culture and 20 µL of milli-Q water to measure the auto-flourescence of the bacteria itself. Positive control contained 180 µL of bacterial culture and 10 µL of milli-Q water and 10 µL of AHL has also been set up to compare the anti-QS effect of different formulations. The plates were incubated in a Spectra Max-M2 Microplate Reader (Molecular Devices, Sunnyvale, CA, USA) at 37 °C. Fluorescence measurements were recorded automatically using a repeating procedure (λ_excitation_ = 480 nm and λ_emission_ = 510 nm, 40 µs, 10 flashes, gain 100, top fluorescence), growth measurements (OD_600_) (λ = 600 nm absorbance filter, 10 flashes) and shaking (5 s, orbital shaking, high speed). The interval between measurements was 60 min. For each experiment, the fluorescence intensity (FI) and OD_600_ values were obtained by subtracting the received values with the fluorescence blank and OD blank above, respectively. All measurements were taken in triplicates.

## 5. Conclusions

In this study, we have developed novel nanocarrier formulations consisting of chitosan and a negatively charged cyclodextrin, Captisol^®^, via the very mild ionotropic gelation technique. The charge ratio ^[+]^/_[−]_ determined the final physicochemical characteristics of the resulting NPs. The nanoparticles exhibited a small size, a positive zeta potential and a great capacity for the association of quercetin. Quercetin-loaded NPs showed to be stable in bacterial M9 medium for 7 h. The presence of Captisol^®^ in the NPs plays an important role in controlling the release rate of quercetin. Chitosan-based NPs can be used as an effective vehicle to deliver hydrophobic bioactive compounds locally to the bacterial surface, as well as to enhance both their anti-QS and anti-bacterial activities. The exact mechanism in which NPs interact with the *E. coli* Top 10 biosensor remains to be elucidated and is currently being addressed in our Laboratory.

## Figures and Tables

**Table 1:** Physicochemical properties of unloaded CS_70/5_/SBEβCD/TPP nanoparticles (mean ± S.D., *n* = 3).

Mass Ratio CS/CD/TPP (*w*/*w*/*w*)	Charge Ratio (^+^/_−_)	Z-Average Size (d. nm)	PDI	ζ-Potential (mV)	Production Yield (%)
4/0/0.75	2.50	413 ± 11	0.15–0.19	+36.9 ± 0.6	69.0 ± 3.2
4/0/0.5	3.75	256 ± 05	0.04–0.10	+36.8 ± 0.8	37.7 ± 1.5
4/1/0.5	2.18	323 ± 18	0.03–0.17	+38.0 ± 0.2	48.3 ± 1.3
4/2/0.25	1.93	259 ± 09	0.04–0.13	+36.3 ± 0.5	45.4 ± 2.0

**Table 2:** Physicochemical properties of unloaded CS_70/20_/SBEβCD/TPP nanoparticles (mean ± S.D., *n* = 3).

Mass Ratio CS/CD/TPP (*w*/*w*/*w*)	Charge Ratio (^+^/_−_)	Z-Average Size (d. nm)	PDI	ζ-Potential (mV)	Production Yield (%)
4/3/0	2.08	378 ± 14	0.16–0.22	+31.0 ± 0.9	40.5 ± 5.7
4/4/0	1.56	602 ± 23	0.23–0.25	+31.5 ± 0.7	62.9 ± 2.1
4/0/1	2.25	335 ± 14	0.19–0.21	+33.9 ± 1.2	55.3 ± 1.9
4/1/0.75	2.03	446 ± 12	0.13–0.25	+40.0 ± 1.1	63.5 ± 2.8
4/2/0.5	1.85	413 ± 31	0.21–0.25	+39.5 ± 1.4	60.1 ± 3.2

**Table 3:** Physicochemical properties of CS_70/5_/SBEβCD/TPP quercetin-loaded nanoparticles (mean ± S.D., *n* = 3).

Mass Ratio CS/CD/TPP (*w*/*w*/*w*)	Charge Ratio (^+^/_−_)	Z-Average Size (d. nm)	PDI	ζ-Potential (mV)	Production Yield (%)
4/0/0.75	2.50	444 ± 08	0.200–0.220	+35.4 ± 1.67	68.77 ± 3.18
4/0/0.5	3.75	316 ± 08	0.062–0.118	+35.5 ± 0.42	49.85 ± 4.92
4/1/0.5	2.18	319 ± 14	0.037–0.131	+37.2 ± 0.45	61.63 ± 5.11
4/2/0.25	1.93	270 ± 05	0.042–0.114	+35.5 ± 0.32	52.86 ± 2.19

**Table 4:** Physicochemical properties of CS_70/20_/SBEβCD/TPP quercetin-loaded nanoparticles (mean ± S.D., *n* = 3).

Mass Ratio CS/CD/TPP (*w*/*w*/*w*)	Charge Ratio (^+^/_−_)	Z-Average Size (d. nm)	PDI	ζ Potential (mV)	Production Yield (%)
4/0/1	2.25	390 ± 24	0.21–0.28	+32.4 ± 1.91	64.24 ± 3.60
4/0/0.75	3.00	485 ± 31	0.30–0.38	+29.3 ± 2.01	54.49 ± 7.88
4/3/0	2.08	332 ± 07	0.17–0.26	+31.5 ± 1.29	45.98 ± 3.48
4/4/0	1.56	572 ± 27	0.22–0.27	+32.8 ± 0.25	68.48 ± 5.21
4/1/0.75	2.03	401 ± 13	0.21–0.24	+35.3 ± 0.92	74.48 ± 6.09
4/2/0.5	1.85	397 ± 21	0.16–0.26	+39.0 ± 0.75	72.69 ± 3.75

## Supplementary Materials

The supplementary materials are available online.
