# Supplementary material for: Chitosan/Cyclodextrin/TPP Nanoparticles Loaded with Quercetin as Novel Bacterial Quorum Sensing Inhibitors
Source: Molecules. 2017 Nov 15;22(11):1975. doi: 10.3390/molecules22111975 (PMC6150374; doi:10.3390/molecules22111975)
Supplement: Supplementary file 1 [file molecules-22-01975-s001.pdf]

molecules

Supplementary Information

Chitosan/Cyclodextrin/TPP nanoparticles loaded with quercetin as novel bacterial quorum sensing inhibitors

Hao Thanh Nguyen[1,3], Francisco M. Goycoolea[1,2,*]

[1] Institute for Biology and Biotechnology of Plants, University of Münster, Münster 48143, Germany; t_nguy32@uni-muenster.de
[2] School of Food Science and Nutrition, University of Leeds, LS2 9JT United Kingdom; F.M.Goycoolea@leeds.ac.uk
[3] Faculty of Biotechnology, Vietnam National University of Agriculture, Vietnam; nthao.cnsh@vnua.edu.vn
*Correspondence: F.M.Goycoolea@leeds.ac.uk; Tel.: +44(0)-113-343-1412

Table S1. Physicochemical properties of unexpected $CS_{70/5}$ NPs. (mean ± S.D., *n* = 3)

Mass ratio CS/CD/TPP (w/w/w)	Charge ratio (+/-)	Size (nm)	PDI	ζ potential (mV)	Production yield (%)
4/1/0	5.20	a	-	-	-
4/2/0	2.60	a	-	-	-
4/3/0	1.73	b	-	-	-
4/4/0	1.30	b	-	-	-
4/5/0	1.04	b	-	-	-
4/6/0	0.87	b	-	-	-
4/1.5/1	1.22	b	-	-	-
4/2/0.75	1.27	b	-	-	-
4/3/0.5	1.19	b	-	-	-
4/4/0.25	1.11	b	-	-	-
4/0/1	1.88	Non-resuspendable	-	-	-

PDI: Polydispersity index; [a] A clear solution was obtained; [b] Precipitation or gel-formation was observed.

Table S2. Physicochemical properties of unexpected $CS_{70/20}$ NPs (mean ± S.D., *n* = 3)

Mass ratio CS/CD/TPP (w/w/w)	Charge ratio (+/-)	Size (nm)	PDI	ζ potential (mV)	Production yield (%)
4/1/0	6.25	a	-	-	-
4/2/0	3.12	a	-	-	-
4/5/0	1.25	b	-	-	-
4/1.5/1	1.46	b	-	-	-
4/2/0.75	1.53	b	-	-	-
4/3/0.5	1.42	b	-	-	-
4/4/0.25	1.33	b	-	-	-
4/0/1.25	1.80	b	-	-	-
4/0/1.5	1.50	b	-	-	-
4/0/2	1.13	b	-	-	-
4/1/1	1.66	b	-	-	-
4/1/0.5	2.62	a	-	-	-
4/2/0.25	2.32	Non-resuspendable	-	-	-

PDI: Polydispersity index; [a] A clear solution was obtained; [b] Precipitation or gel-formation was observed.

Table S3. The effect of Captisol® concentration on the solubility of quercetin, encapsulation efficiency (EE) and the final loading of quercetin into $CS_{70/5}$ nanoparticles (mean ± SD, *n* =3). Control values: 4/0/0.75 NPs

Mass ratio CS/CD/TPP (w/w/w)	Charge ratio (+/-)	Captisol® conc. (mg/mL)	Quer. Solubility conc. (mg/mL)	Association efficiency (%)	Loading efficiency (%)
4/0/0.75	2.5	0	0.0095 ± 0.0004	95.38 ± 0.67	0.50 ± 0.0335
4/0/0.5	3.75	0	0.0095 ± 0.0004	96.80 ± 0.76	0.55 ± 0.0236
4/1/0.5	2.18	0.375	0.0219 ± 0.0013	93.20 ± 1.36	0.98 ± 0.0624
4/2/0.25	1.93	0.75	0.0394 ± 0.0009	90.45 ± 1.30	1.49 ± 0.0252

Table S4. The effect of Captisol® concentration on the solubility of quercetin, encapsulation efficiency (EE) and the final loading of quercetin into $CS_{70/20}$ nanoparticles (mean ± SD, *n* =3). Control values: 4/0/1 NPs

Mass ratio CS/CD/TPP (w/w/w)	Charge ratio (+/-)	Captisol® conc. (mg/mL)	Quer. Solubility conc. (mg/mL)	Association efficiency (%)	Loading efficiency (%)
4/0/1	2.25	0	0.0083 ± 0.0004	67.29 ± 3.90	0.30 ± 0.0159
4/0/0.75	3	0	0.0083 ± 0.0004	71.43 ± 2.43	0.33 ± 0.0112
4/3/0	2.08	1.125	0.0646 ± 0.0051	91.82 ± 2.07	2.20 ± 0.1078
4/4/0	1.56	1.5	0.0787 ± 0.0018	94.69 ± 0.33	2.43 ± 0.0084
4/1/0.75	2.03	0.375	0.0219 ± 0.0013	85.42 ± 2.28	0.86 ± 0.0230
4/2/0.5	1.85	0.75	0.0394 ± 0.0009	85.17 ± 3.25	1.35 ± 0.0404

Table S5. Influences of different formulations on the reduction of bacterial growth (OD_{600}) and relative QS activities as compared to the positive control

**Free components**	**Captisol 0.1875 mg/mL**	**Captisol 0.375 mg/mL**	**Captisol 0.75 mg/mL**	**$CS_{70/5}$ 0.75 mg/mL**	**$CS_{70/20}$ 0.75 mg/mL**	**Quercetin 0.0125 mg/mL**	**Quercetin 0.025 mg/mL**	**Quercetin 0.0375 mg/mL**
OD reduction	-2.09%	0.48%	1.18%	21.08 %	88.67 %	25.38%	29.06%	33.34%
QS reduction	8.43%	17.53%	20.86%	27.08 %	65.79 %	-54.95%	-35.57%	-20.97%
**$CS_{70/20}$ NPs**	**U 4/0/1**	**U 4/2/0.5**	**U 4/3/0**	**U 4/4/0**	**L 4/0/1**	**L 4/2/0.5**	**L4/3/0**	**L 4/4/0**
OD reduction	30.10%	71.33%	44.64%	63.34 %	39.48 %	81.96%	60.03%	88.32%
QS reduction	43.36%	69.27%	64.08%	78.57 %	51.46 %	72.93%	78.43%	100.00%
**$CS_{70/5}$ NPs**	**U 4/0/0.75**	**U 4/1/0.5**	**U 4/2/0.25**	**L 4/0/0.75**	**L 4/1/0.5**	**L 4/2/0.25**	U: unloaded L: loaded NPs: nanoparticles	
OD reduction	21.80%	22.72%	21.14%	25.18 %	30.33 %	34.71%		
QS reduction	30.90%	38.45%	38.07%	38.18 %	52.32 %	61.12%		

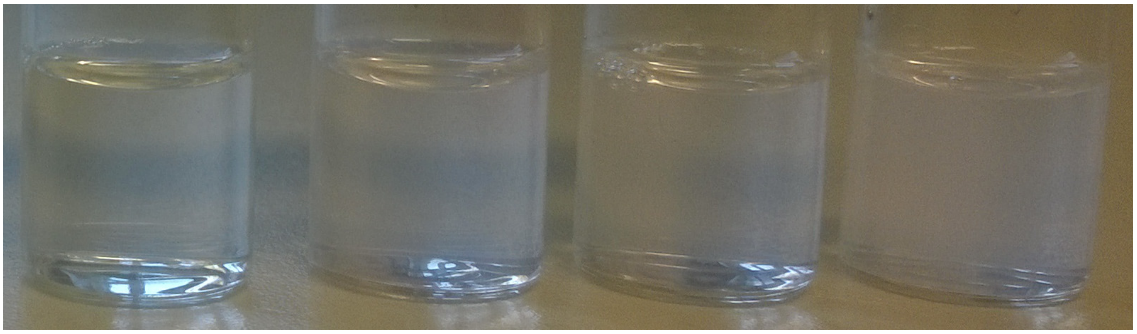

4/0/0.5 **4/0/0.75** **4/1/0.5** **4/2/0.25**

Figure S1. Unloaded CS$_{70/5}$ nanoparticles

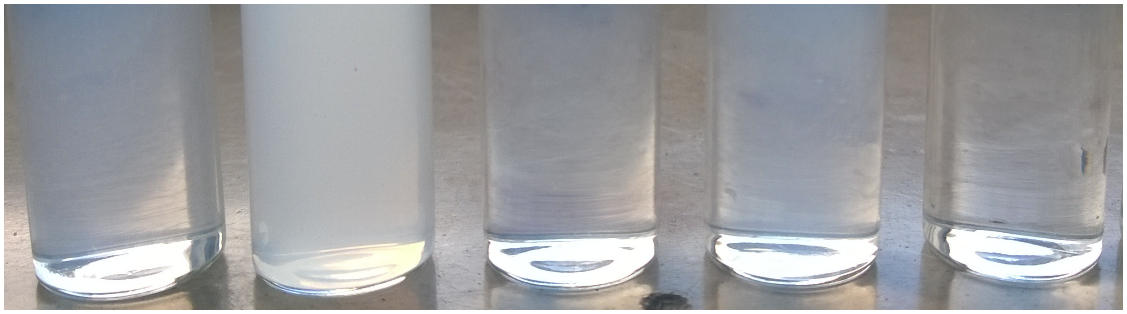

4/3/0 **4/4/0** **4/0/1** **4/2/0.5** **4/1/0.75**

Figure S2. Unloaded CS$_{70/20}$ nanoparticles
